# Chinese EFL Students' Perceptions of Classroom Justice: The Impact of Teachers' Caring and Immediacy

**DOI:** 10.3389/fpsyg.2021.767008

**Published:** 2021-10-21

**Authors:** Ping Yan

**Affiliations:** School of Foreign Languages, Henan University, Kaifeng, China

**Keywords:** classroom justice, teachers' caring, teachers' immediacy, English as a foreign language (EFL), students' perception

## Abstract

The correlation between students' perceptions of three dimensions of classroom justice, teacher immediacy, and teacher caring has been found important since it can provide a learning ambiance for students in which they can enthusiastically learn a new language. To find out this relationship, the present study has strived to probe into the interplay between the aforementioned variables and to see whether teacher caring and teacher immediacy can predict students' perception of justice. In so doing, the participants of this study were 1,178 Chinese EFL students of various ages and education levels. Three instruments were utilized in this study to perceive the students' perception of classroom justice, teacher immediacy, and teacher caring. To collect the data, these instruments were distributed through an online survey software called *Wenjuanxing* (Questionnaire Star). Results demonstrated that there was a positive association among these three variables, and utilizing SEM analyses, it was found that both teacher immediacy and teacher caring predict students' perception of classroom justice that implies teachers who are found to be more caring and give appropriate verbal and non-verbal immediacy where needed, are perceived to change the students' understanding of the classroom justice to a positive attitude. Finally, the results of this study were discussed regarding previous findings, and accordingly, some implications were put forward in the EFL context.

## Introduction

Teachers without a shadow of a doubt are said to be one of the most important stakeholders particularly in the English as foreign/second language contexts and by whom students' perception of justice and engagement are highly impacted (Pishghadam et al., 2019). It was positive psychology that paves the way to think of the teacher-student relationship as a humanistic concept and it has been claimed that the way students are treated in the classroom affects their learning process. Furthermore, in order to enhance teacher quality, some strategies should be implemented to heighten students' passion and cause them to actively engage in the activities (MacIntyre and Mercer, 2014). Students' perception of classroom justice is of paramount importance as well. As it was claimed by Ciuladiene and Račelyte (2016), three types of justice can be dealt with considering the educational context. Distributive justice refers to the grades which are received by the students and the amount to which students attract their teachers' attention. The second category of justice is procedural justice which incorporates how students' homework is evaluated, what methods are used by the teacher to manipulate the class, and the strategies applied in the class for students' behavior to be controlled. The third category of justice in the classroom is instructional justice. It is concerned with how much students are treated reverently and courteously by the teachers, and if the information can be clearly conveyed to the students by the teacher.

Another point that can be emphasized is teacher care and teacher immediacy through which worthwhile engagement can be created. Teacher care as a strategy falls under the category of teacher clarity in that the clearer the teacher's words, teaching methods, and examples are, the higher the comprehension and engagement would be and accordingly, it builds up an excellent teacher-student rapport. Teacher immediacy is described as a collection of non-verbal and verbal strategies and behaviors that are given by the teachers to generate a sense of closeness with the students (Cakir, 2015; Xie and Derakhshan, 2021). Moreover, studies show that teacher clarity and immediacy are interconnected; however, their functions are a bit different (Titsworth et al., 2015). They are at alteration since teacher clarity causes cognitive enthusiasm and teacher immediacy provokes emotional passion (Mazer, 2013). Both teachers' caring and teachers' immediacy are relevant to positive psychology in which the focus puts on how individuals can thrive and be happier, concentrating on positive emotions like hope, enthusiasm, resilience, positivity, and so forth rather than negative feelings (Dewaele, 2015; MacIntyre et al., 2019) which means when students are behaved fairly in the class by their teachers through being given care as well as verbal and non-verbal immediacy, it boosts students' spirits and makes them feel better about themselves. The current study has stressed the predictive impact of teachers' caring and immediacy on students' perception of justice. It is of great importance that how students are treated in the class shapes their viewpoints about the classroom and it strongly impacts teachers themselves; therefore, the association among these three variables has been dealt with in this study. It differs from the previous studies in two aspects. Firstly, even though knowing the correlation among students' perception of justice, teacher caring, and teacher immediacy reveals benefits that can be practiced by teachers to have a nice learning environment, reviewing the relevant studies presented that no experimental study has tested the concurrent effects of these three variables so far. For instance, a study carried out by Liu (2021) showed that students' motivation is positively impacted by teacher immediacy. Despite the fact that it was a comprehensive study, just one of the variables in the present study was used there. Another example that can be taken into account is a study carried out by Zheng (2021) in which five other variables were dealt with: teachers' clarity, immediacy, credibility, students' motivation, and students' engagement. Therefore, these three variables in the current study have not been analyzed in previous studies. Secondly, this study has been conducted in China which is another factor that makes it different from the previous studies. In an effort to find the relationship between these three variables, the author in the present research made an endeavor to answer the next two research questions:

1. Are there any significant relationships between Chinese EFL teachers' caring, immediacy, and their students' perceptions of classroom justice?
2. Do Chinese EFL teachers' caring and immediacy significantly predict their students' perceptions of classroom justice?

## Background

### Classroom Justice

#### The Importance of Justice in Life and in Educational Contexts

Justice is of great importance in life; it can be seen in political sciences, social sciences, and organizational behaviors in society. Other factors are related to justice in society and it is why it attracted much attention (Cropanzano and Greenberg, 1997). Regarding the educational contexts, many famous people have established the social psychology theory of justice. It has been discovered that many student variables are impacted by students' perception of classroom justice such as the students' level of motivation, academic aims, engagement, the teacher-student relationship, both behavioral and emotional reactions, showing enthusiasm in the subjects, the amount of willingness to talk, how they feel about their teacher, and cognitive learning. Positive equity, inspiration, and affective learning are decidedly anticipated by perceptions (Chory-Assad, 2002), and they positively affect accomplishment (Burns and DiPaola, 2013). Many studies of justice in the instructional context have concentrated on the students' perceptions of classroom justice that are related to their behavioral/affective answers and academic results (Rasooli et al., 2018). Nonetheless, how teachers identify their own justice in different aspects of the classroom is critical for creating and keeping a just classroom environment (Grazia et al., 2020). Students' perception of unfairness in the context of the class can cause negative behavior, fight, dishonesty, anger, and struggle increase as shown by Ciuladiene and Račelyte (2016). This study discovered students who experience unjust teacher behavior in the learning contexts. It was reported by the students in the mentioned study that they experienced interactional injustice more often than experiencing distributive or procedural injustice.

#### Distributive Justice

Justice can be categorized into three categories: distributive, procedural, and interactional justice. Distributive justice is described as an understanding of fairness regarding distributing outcomes. Three following principles can explicate this type of justice more: need (how the outcome is distributed when one expects or needs something), equity (how the outcome is distributed according to one's diligence, contribution, and performance), and equality (how equally the outcome among people are distributed) (Deutsch, 1975). School was portrayed by Dalbert and Stoeber (2006) as a space where students face significant distribution choices. Distributive equity can be dissected regarding the grades which are gotten by various students and with the point that who stands out enough to be noticed by the teacher (Chory-Assad and Paulsel, 2004).

#### Procedural Justice

Procedural justice refers to the understanding of fairness in terms of utilized measures and policies so as to make allocation decisions. This justice is perceived to be kept when the measures are judged to be fair such as “bias dominance principal,” recognized on satisfactory and accurate information such as “accuracy principle,” engaged regularly across time and individuals such as “consistency principle,” adaptable such as “correctability principle,” considering all individuals' concerns who are engaged such as “voice principle,” resting on the predominant ethical and moral values such as “ethicality principle”, enacted clearly and with clarity such as “transparency principle,” and are rational “reasonableness principle” (Leventhal, 1980; Kazemi and Törnblom, 2008; Rasooli et al., 2019). Interactional and procedural fairness are inspected in the instructive contexts as well. It was mentioned by Chory-Assad (2007) that there are three cycles in the instructional contexts, regarding procedural equity. The main cycle incorporates the manners in which homework assignments are evaluated, the second includes techniques utilized by the teacher to run the class, and the third deals with the arrangements used to control the students' conduct. Fair techniques that can be used to lead the class are clarified in Horan et al. (2010). Nine classifications were grouped thinking about procedural unfairness. Just as evaluating techniques, it is additionally worried about different issues, for example, cosmetics/late arrangements, planning, data for tests, criticism, teachers' mistakes, not finishing promises, class methods, and not upholding strategies. This idea was upheld by Houston and Bettencourt (1999) that upheld the idea that justice manages activities in regards to the clearness and precision of the information offered to the students with respect to the class and tests.

#### Interactional Justice

Interactional justice that is the third classification of organizational justice, pertains to the understanding of fairness in conveying information and interpersonal relationships, when individuals perceive to be in a friendly atmosphere such as “caring principle,” behaved reverentially such as respect principle, and with dignity such as “propriety principle,” and when information is transferred to them in an appropriate manner such as “timeliness principle,” sincerely such as “truthfulness principle,” and according to sufficient and rational clarifications such as “adequacy/justification principle” (Greenberg, 1993; Colquitt, 2001; Rasooli et al., 2019). Out of the three dimensions, distributive justice was the one which was said to be the most crucially important from the teacher's point of view which can be seen in different forms, from grading, giving feedback to praising and providing students with opportunities. Interactional equity alludes to the justice and quality of interpersonal treatment that people are given when strategies are cultivated.

As indicated by Chory-Assad (2002), interactional equity incorporates two elements. The principal thing is passing on data obviously. The subsequent one is concerned with dignity and reverence. In the classroom, interactional equity is concerned with the degree to which educators connect with their students politely, honorably, and straightforwardly. Assessments of the teacher's interactional equity concern when the educator care about students' perspectives, pays attention to their interests and interacts in a decent way with them Chory-Assad and Paulsel (2004). Horan and associates' examination recommended that interactional equity issues included harshness/discourteousness, expressing or suggesting ineptitude, racist and biased comments, singling out understudies, blaming students for bad behavior, and teacher affecting intellectually (Estaji and Zhaleh, 2021).

### Teacher Caring

Teacher care was first introduced by Noddings (1984) which refers to the amount of empathy shared and the openness in face of other people's needs. Likewise, the same goes for the educational contexts (Gasser et al., 2018). Students' well-being, feeling revered, the amount of being engaged during the class, the level of self-esteem, and their performance are stimulated when teachers show care (Derakhshan et al., 2019). In a study conducted by Liu et al. (2021), the relationship between teacher support, which can be regarded as a part of teachers' caring, and creative self-efficacy with students' autonomous motivation and achievement motivation was studied in China. It has been found that students' motivation in academic contexts was affected by teachers' support; however, no considerable association was discovered between teachers' support and students' creative self-efficacy. Although this study was of great importance due to the variables analyzed, the present study can be noticeable since it has put forward three main variables, especially teachers' caring which can be perceived as an umbrella term for teacher support. Teachers' caring is a substantial part of the educational context since it causes students to feel revered and they are more inclined to accept the classroom values in this way. When classroom values are respected by the students, they are more likely to learn the materials in a better way. It was claimed that caring about others makes one care about himself as well (Ryan and Deci, 2000; Ware, 2006). As indicated by Mayeroff (1971), caring is an interaction through which one becomes more acquainted with someone else, thinking about earlier conduct, patience, truthfulness, quietude, and dependence. Mayeroff (1971) suggests that caring is not necessarily a reciprocal act. Similarly, Bluestein (1991) perceives that a relationship comprises of specific jobs that may not include corresponding practices. For example, the relationship between instructors and students can be characterized by a role that the instructor should really focus on students as his responsibility in his expert work. Albeit caring has been related to progress, secondary school students' understanding is restricted, and it is perhaps because of the way that their theoretical reasoning has not been formed at this point. It was accentuated in past examinations that students were almost certain to remain in school when they saw educators as just (Knesting, 2008). Instructor caring was shown by practices that worked on students' gifts, support their confidence, value their thoughts, and revere them as people. Likewise, Geary (1988) suggested that school achievement was facilitated by an instructor who is caring, approachable, compatible, encouraging, considerate, a decent audience, and funny. In a thorough report, Coburn and Nelson (1989) studied around 300 Native-American secondary school students in Washington, Montana, Oregon, and Idaho. These students portrayed efficacious teachers as gracious, mindful, listening altogether, showing an uplifting perspective, giving assistance promptly, reassuring, agreeable, having the students engaged in activities, and giving students positive feedback.

In addition, Coley (1995) investigated information from the National Center for Education Statistics to perceive factors that brought about the drop-out rate in the United States. It was accounted for that about 43% of students surrendered school since they loathed school; under 40% told that getting grades that were not palatable was the reason; over 25% expressed there was a phony connection between their instructors and them, and about 25% could not feel a sense of having a place at school.

### Teacher Immediacy

Teacher Immediacy is conceptualized as a range of behaviors such as giving a smile, making eye contact, and close premixes which help the communicators to form a sense of physical and psychological closeness (Richmond et al., 2008). Verbal immediacy can be exemplified in this way: when students are asked about their ideas, they are asked to be involved in a friendly conversation, and teachers use a great sense of humor. Non-verbal immediacy, on the other hand, refers to teachers smiling, making eye contact, and using relaxing postures (Wendt and Courduff, 2018). It has been shown that students' empowerment and engagement can be enhanced through teacher immediacy, their anxiety decreases, and their attention is sustained (Bolkan, 2017). In a study conducted by Derakhshan (2021) both language teachers' non-verbal immediacy and credibility have been found to be predictors of Turkman students' academic engagement. Gholamrezaee and Ghanizadeh (2018) also tested how students' self-actualization, self-esteem, stress-control, cognitive learning, and emotional exhaustion have been affected by EFL teachers' immediacy. It has been discovered through the SEM analysis that all the constructs relevant to the students, particularly students' self-actualization were positively impacted by teacher immediacy. Similarly, according to Sheybani (2019), students' willingness to communicate was significantly and positively influenced by teachers' verbal and non-verbal immediacy.

Teacher immediacy can be exemplified in different forms such as using out-of-the class examples and experiences so as to create a sense of closeness and cause students to fit in with a new language which is actually viewed as a new culture. Asking questions and encouraging students to talk that causes them to start talking in a foreign/second language regardless of all the language barriers which can be experienced while learning a new one. Using humor in class brings about many advantages, out of which is establishing a friendly atmosphere in which students are not horrified by speaking even if they are panic-stricken. Addressing students by name is another behavior that can be used by the teacher to increase a sense of value in students and as a result, they may feel they have the bravery to start talking due to the fact that they may make some mistakes. Parsing students' work, actions, or comments falls under the category of verbal immediacy that has been found to play a paramount role in students' well-being and their academic achievements. Considering both types of teacher immediacy, the following examples are perceived as non-verbal immediacy. Sitting behind the desk while teaching is said to be monotonous for students and they may lose concentration while their teacher gets stuck at his desk. Smiling at individual students in the class is another construct that can be practiced by teachers if they are inclined to build up a nice rapport with the students. Having a very tense body position, additionally, causes a stressful situation and makes students feel fearful to be engaged in the activities.

### Positive Psychology

A new era of positive psychology happened in the educational context when MacIntyre and Gregersen (2012) put emphasis on it. Therefore, researchers' concentration was shifted from negative emotions such as anxiety and boredom (Marcos-Llinás and Garau, 2009; Pawlak et al., 2020; Derakhshan et al., 2021) to both negative and positive emotions (Kruk, 2021) that can be found in the learning and teaching process. It was highlighted in applied positive psychology that both negative and positive emotions are interwoven, and they cannot be separated from each other in many contexts and sometimes they are complementary (MacIntyre and Gregersen, 2012; Wang et al., 2021). Positive emotions have been said to add more meaning and enjoyment to the process of learning, and it causes students to be more resilient when encountering challenging issues in the instructional context (Gregersen, 2013).

Positive psychology dramatically throve in 2016 after a thorough book published by MacIntyre et al. (2016). In their book, the authors noted that four main contributions have been made by positive psychology that impacted L2 education. The first one emphasized the movement from negative emotions to positive emotions which means emotions will play a pivotal role in L2 educational contexts, and both teachers and the educational achievements of the students (Li, 2020). The second noticeable influence of positive psychology in instructional contexts is the model which was entitled as “model of character strength” (Park et al., 2004). Six categories of characters are found to have a paramount impact on personal development: fairness, superiority, humanity, moderation, bravery, and wisdom. When it comes to an educational concept, provided that these characteristics are strengthened by teachers and learners, they can thrive (MacIntyre, 2021). The third influence is the movement from PERMA to EMPATICS to perceive well-being within positive psychology (Oxford, 2016). It was Seligman (2011) who devised the PERMA model l, which is a controversial concept, stands for Positive emotions, Engagement, Relationships, Meaning in life, and Accomplishment. According to the model raised, in order to find meaning in life, a strong positive connection should be among these factors resulting in individuals' well-being (Mercer and Gregersen, 2020). After that, this model was expanded by Oxford (2016, p. 10) which was called: EMPATHICS, incorporating the nine components of “(1) Emotion and empathy, (2) Meaning and motivation, (3) Perseverance, including hope, resilience, and optimism, (4) Agency and autonomy, (5) Time, (6) Hardiness and habits of mind, (7) Intelligences, (8) Character strengths, and (9) Self factors self-verification, self-esteem, self-concept, and self-efficacy.” As indicated by Oxford (2016) many of these factors such as empathy and resilience have not been studied by researchers yet which means they can be utilized for further research. The fourth influence of positive psychology in L2 contexts is flow theory (Csikszentmihalyi, 1990) that is the extent to which one is so immersed in doing tasks that he forgets about time. Reference as a result, students' L2 learning attainments, and success are highly influenced by students' experiencing flow.

It has been said that students' perceptions of classroom justice have a positive correlation with both teacher caring and teacher immediacy. It is perceived by the students as fair when they are treated as follows: being given enough attention by their teachers, being provided with feedback on one's individual work, using comments on papers or oral discussions, being addressed by their first name, and being asked some questions, and being encouraged to talk which fall under the category of verbal immediacy. On the other hand, using a dull voice when talking to class, sitting on a desk or in a chair while teaching, and having a very tense body position while talking to the class that falls under the category of non-verbal teacher immediacy are viewed as unfair by the students. In terms of teacher caring, when a teacher is understanding and sympathetic, they are viewed as fair teachers by the students. As has been revealed by Greenier et al. (2021), there is a positive correlation between teachers' psychological well-being and their work engagement. Therefore, when a teacher feels good about his personal and working life, he can be actively engaged in what he does and as a consequence, they energize students to be involved in the activities.

## Methods

### Participants' Demographic Information

In this study, the final 1,178 participants were from four universities in Henan province, namely Henan University, Henan Polytechnic University, Zhengzhou University of Aeronautics and Huanghuai University. To maximize the variation of the sample that enhances the generalizability of the outcomes, participants were from more than 15 majors including Chinese literature, French language, Law, Philosophy, Chemistry, Biological Sciences, Accounting, etc. They were heterogeneous in terms of gender, with 342 (29.03%) male and 830 (70.46%) female, six participants (0.51%) preferred not to reveal their gender identity. In the sample, participants were of different levels of education, with 266 (22.58%) freshmen, 838 (71.14%) sophomores, 56 (4.75%) juniors, and 18 (1.53) seniors. They were opted for based on random sampling. The respondents who were reassured that their information would be kept secret and be utilized only for research purposes signed their consent agreement before they participated in this survey.

### Instruments

#### Students' Perceptions of Classroom Justice

The present study drew on Chory-Assad and Paulsel's (2004) scale to evaluate students' comprehension of classroom justice. It includes distributive justice that contains two parts with 14 items on which students were expected to evaluate the fairness of the grades given in a course. It also encompasses procedural justice including 17 items on which students were supposed to evaluate teachers' policies and schedules, and it also contains interactional justice including 8 items on which students were expected to evaluate teachers' interpersonal relationship with their students. Three examples for the above instruments are, respectively, as follows: students' grades on the last exam were compared to the ones of their classmates; how the teacher coordinates the class discussions, and the way students are treated by their teachers. All the aforementioned instruments were supposed to be rated on a 5-point Likert Scale ranging from extremely unfair (1) to extremely fair (5).

#### Teachers' Caring

The other instrument used in this study was teacher caring developed and validated by Koehn and Crowell (1996). The items were introduced in a standard way and each item included a seven-step continuum for the response. Students were supposed to rate the items and indicate their feeling about their current teacher in the following way: Numbers 1 and 7 showing a very strong feeling, numbers 2 and 6 indicating a strong feeling, numbers 3 and 5 expressing a fairly weak feeling, and number 4 showing the students are undecided.

#### Teachers' Immediacy

The subsequent instrument estimated students' impression of teacher immediacy developed by Richmond et al. (1987), featured two parts, the first part that evaluated teachers' verbal immediacy includes 17 items, and the second part that evaluated teachers' non-verbal immediacy consists of five items. Students were supposed to rate the items on a 5-point Likert Scale ranging from Never (0) to almost always (4).

### Data Collection Procedure

The questionnaires mentioned above consists of three sections and 71 items in total. To assure the accurate understanding of the questions and credibility of responses, all the instructions and items were conducted in Chinese. A free online survey platform called *Wenjuanxing (Questionnaire Star)* was utilized to generate the electronic questionnaire.

The link of questionnaire was sent to English teachers and tutors of above mentioned four universities through Wechat, and then was sent to class Wechat group. Students may feel free to fill the questionnaire if they were willing to participate in the survey. The survey was conducted between July 21 and July 22. The final 1,178 participants were of different levels of education, with 266 (22.58%) freshman, 838 (71.14%) sophomore, 56(4.75%) junior, and 18 (1.53) senior. They were from different colleges, covering more than 15 majors. The respondents who were reassured that their information would be kept secret and be utilized only for research purposes gave consent as the first item of electronic questionnaire. Their personal information would remain confidential.

### Data Analysis

In the present study, to find the relationship between the students' perception of classroom justice, teacher caring, and teacher immediacy, Pearson correlation was utilized which showed that all the sub-constructs of classroom justice are positively correlated with both teachers' caring and immediacy. Likewise, SEM analysis was used to find if Chinese EFL teachers' caring and immediacy significantly predict their students' perceptions of classroom justice. It has been shown that students' perceptions of classroom justice are significantly predicted by both Chinese EFL teachers' caring and immediacy.

## Results

Table 1 illuminates the normality of the data, utilizing Kolmogorov-Smirnov test.

**Table 1:** The results of K-S test.

	**Kolmogorov-Smirnov^a^**		
	**Statistic**	***df***	**Sig**.
Classroom justice	0.08	1,178	0.09
Teachers' caring	0.09	1,178	0.08
Teachers' immediacy	0.06	1,178	0.11

a *Lilliefors significance correction*.

The results of the Kolmogorov-Smirnov test indicated that the data are normally distributed across all variables and parametric statistics can be utilized. Table 2 displays descriptive statistics of Chinese EFL teachers' caring, immediacy, and their students' perceptions of classroom justice including the number of participants, the mean, and the standard deviation.

**Table 2:** Descriptive statistics of the variables of the study.

	***N***	**Minimum**	**Maximum**	**Mean**	***SD***
Classroom justice	1,178	66	195	170.96	15.50
Teachers' caring	1,178	31	70	52.10	8.08
Teachers' immediacy	1,178	13	88	60.16	10.35

As Table 2 shows, 1,178 students participated in the present study. Besides, it was found that classroom justice has a mean score of 170.96, teachers' caring has a mean score of 52.10, and teachers' immediacy has a mean score of 60.16. Table 3 summarizes the information obtained from Cronbach alpha analyses.

**Table 3:** Results of Cronbach alpha indexes.

**Scale**	**Subscales**	**Cronbach alpha**
Teachers' caring		0.82
	Distributive1	0.85
Classroom justice	Distributive2	0.91
	Procedural	0.95
	Interactional	0.89
	Overall justice	0.96
	Verbal	0.94
Teachers' immediacy	Non-verbal	0.70
	Overall immediacy	0.94

As can be seen, the utilized questionnaires gained acceptable indexes of Cronbach alpha as a whole as well as in their subscales.

To answer the first research question, Pearson correlation was employed. Table 4 shows the results of Pearson correlation between overall EFL teachers' caring, immediacy, and their students' perceptions of classroom justice.

**Table 4:** Results of Pearson correlation between overall EFL teachers' caring, immediacy, and their students' perceptions of classroom justice.

		**Justice**	**Caring**	**Immediacy**
Justice	Pearson correlation	1		
	Sig. (2-tailed)			
	*N*	1,178		
Caring	Pearson correlation	0.56^**^	1	
	Sig. (2-tailed)	0.000		
	*N*	1,178	1,178	
Immediacy	Pearson correlation	0.50^**^	0.48^**^	1
	Sig. (2-tailed)	0.000	0.000	
	*N*	1,178	1,178	1,178

** *Correlation is significant at the 0.01 level (2-tailed)*.

As it can be seen in Table 4, there are positive significant relationships between overall teachers' caring and students' perceptions of classroom justice (*r* = 0.56, *n* = 1,178, *p* = 0.000, α = 0.01) and their immediacy (*r* = 0.48, *n* = 1,178, *p* = 0.000, α = 0.01). Moreover, there is a positive significant relationship between overall teacher immediacy and students' perceptions of classroom justice (*r* = 0.50, *n* = 1,178, *p* = 0.000, α = 0.01).

Table 5 shows the results of the Pearson correlation between all sub-constructs students' perceptions of classroom justice and overall teachers' caring.

**Table 5:** Results of Pearson correlation between all sub-constructs students' perceptions of classroom justice and overall teachers' caring.

	**Distributive1**	**Distributive2**	**Procedural**	**Interactional**
Teachers' caring	0.47^**^	0.50^**^	0.55^**^	0.56^**^

** *Correlation is significant at the 0.01 level (2-tailed)*.

As Table 5 demonstrates, there are positive significant relationships between all sub-constructs students' perceptions of classroom justice and overall teachers' caring: Distributive1 (*r* = 0.47, *n* = 1,178, *p* = 0.000, α = 0.01), Distributive2 (*r* = 0.50, *n* = 1,178, *p* = 0.000, α = 0.01), Procedural (*r* = 0.55, *n* = 1,178, *p* = 0.000, α = 0.01), Interactional (*r* = 0.56, *n* = 1,178, *p* = 0.000, α = 0.01).

Table 6 shows the results of Pearson correlation between all sub-constructs students' perceptions of classroom justice and overall teachers' immediacy.

**Table 6:** Results of Pearson correlation between all sub-constructs students' perceptions of classroom justice and overall teachers' immediacy.

	**Distributive1**	**Distributive2**	**Procedural**	**Interactional**
Verbal	0.42**	0.44**	0.49**	0.50**
Non-verbal	0.36	0.36	0.40	0.41

** *Correlation is significant at the 0.01 level (2-tailed)*.

As Table 6 demonstrates, there are positive significant relationships between all sub-constructs students' perceptions of classroom justice and verbal immediacy: Distributive1 (*r* = 0.42, *n* = 1,178, *p* = 0.000, α = 0.01), Distributive2 (*r* = 0.44, *n* = 1,178, *p* = 0.000, α = 0.01), Procedural (*r* = 0.49, *n* = 1,178, *p* = 0.000, α = 0.01), Interactional (*r* = 0.50, *n* = 1,178, *p* = 0.000, α = 0.01). Furthermore, there are positive significant relationship between all sub-constructs students' perceptions of classroom justice and non-verbal immediacy: Distributive1 (*r* = 0.36, *n* = 1,178, *p* = 0.000, α = 0.01), Distributive2 (*r* = 0.36, *n* = 1,178, *p* = 0.000, α = 0.01), Procedural (*r* = 0.40, *n* = 1,178, *p* = 0.000, α = 0.01), Interactional (*r* = 0.41, *n* = 1,178, *p* = 0.000, α = 0.01).

**Figure 1:**
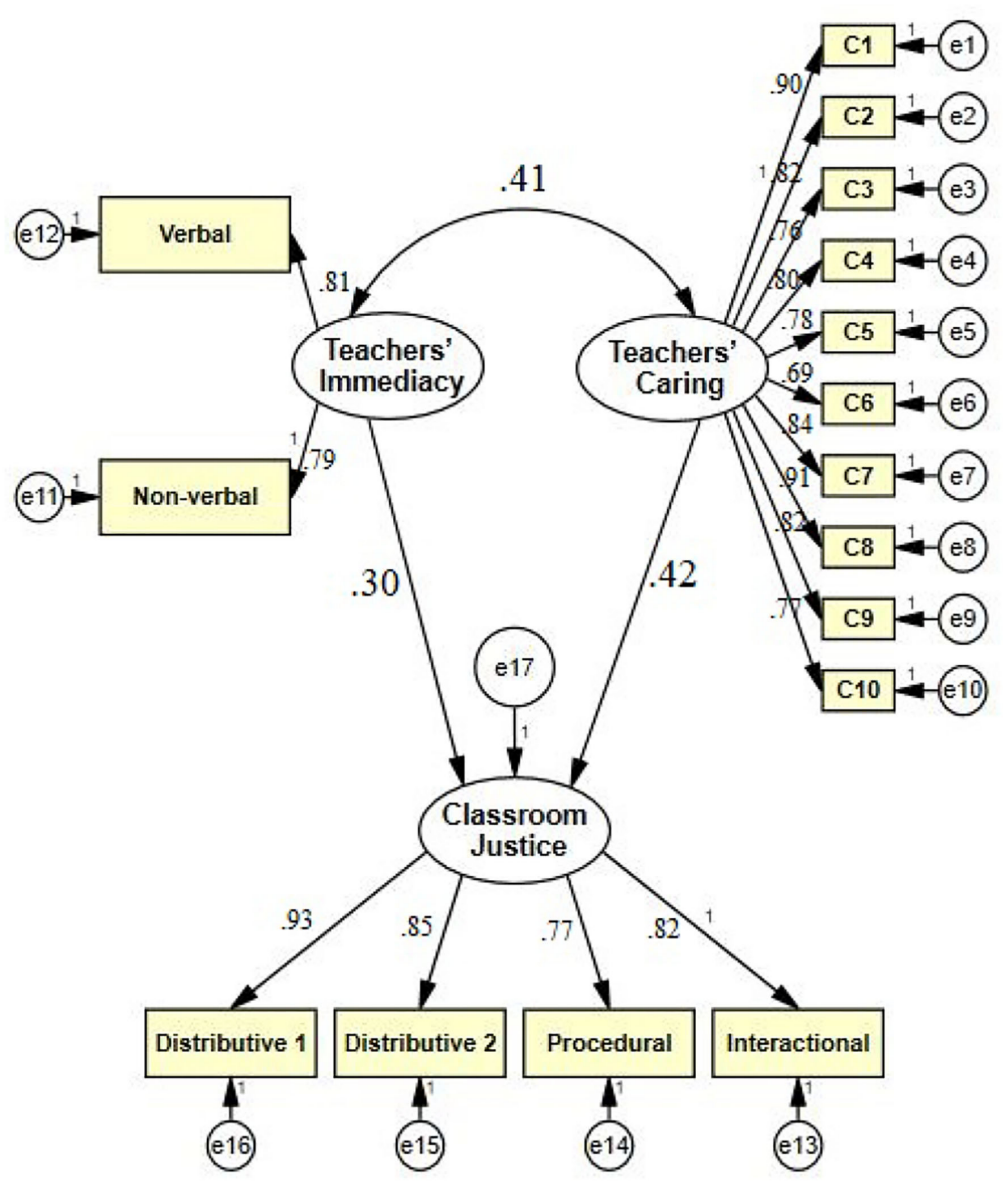
The model of the interrelationships among variables.

To address the second research question, SEM was used through Amos 24. For the qualities of the causal connections among the segments to be checked, the normalized estimates were analyzed. As shown in Figure 1, the model of the interrelationships among factors.

As indicated in Figure 1, both teachers' immediacy (β = 0.30, *p* < 0.05) and teachers' caring (β = 0.42, *p* < 0.05) are positive significant predictors of students' perceptions of classroom justice. Finally, teachers' immediacy correlated positively and significantly with their caring (β = 0.41, *p* < 0.05).

For the model fit to be checked, goodness of fit indices was utilized. Goodness of fit indices can be visible in Table 7. In this study, χ^2^/*df* , GFI, CFI, and RMSEA were employed. In order to have a fit model, χ^2^/*df* is required to be <3, GFI CFI, and NFI is required to be above 0.90, and RMSEA is required to be <0.08.

**Table 7:** Goodness of fit indices.

	***X*^2^/*df***	**GFI**	**CFI**	**NFI**	**RMSEA**
Acceptable fit	<3	>0.90	>0.90	>0.90	<0.08
Model	2.15	0.94	0.95	0.93	0.07

Table 7 delineates that all the integrity of fit indices can run inside the satisfactory level. Thus, the model had a reasonable level of validity.

## Discussion

The current study aimed to probe into a predictive role of EFL teachers' caring and teachers' immediacy in students' perception of classroom justice that was done in China. Some important findings were put forward through this research. Among the variables, classroom justice has the highest mean, while teachers' caring obtained the lowest mean. As can be implied, the highest correlation is ascribed to teachers' caring and students' perception of classroom justice. The second highest correlation can be seen between teachers' immediacy and students' perception of classroom justice. It is also found that the relationship between all the sub-constructs of classroom justice is positive and out of which interactional justice reached the highest (*r* = 0.56, *p* = 0.000). Considering all the sub-factors of classroom justice, it can be perceived that all the sub-constructs including distributive, procedural, and instructional justice are positively correlated with the sub-constructs of teachers' immediacy including verbal immediacy and non-verbal immediacy, among which the highest relationship can be found between instructional justice and verbal immediacy. It implies that teachers' caring and teachers' immediacy have a pivotal impact on how students perceive classroom justice. If the students are supported and are given verbal and non-verbal immediacy, their perception of classroom justice will meaningfully enhance. Last but not least, the SEM analysis clearly showed that both teachers' immediacy and teachers' caring are positive significant predictors of students' perceptions of classroom justice.

The current study aimed to test a predictive role of teacher immediacy and teacher care in Chinese students' perception of justice. The importance of three variables, students' perception of classroom justice, teacher immediacy, and teacher caring as well as the relationship among the variables have been discussed. As indicted by Pishghadam et al. (2019) productive language teaching is something beyond just teaching the subject-matter and conveying information, yet, the psychological aspect of teaching in the educational area should be emphasized. Since teachers and students communicate well in a friendly learning ambiance, it seems essential that a nice rapport be shaped (Xie and Derakhshan, 2021). The results revealed that these three variables were positively correlated. Classroom justice is perceived as positive through students' eyes provided that they are treated with care and also with appropriate verbal and non-verbal immediacy by their teachers.

With regard to the first research question of this study, the results of correlational analyses showed that there is a significant positive correlation, first, between teacher immediacy and students' perception of classroom justice, second, between teacher caring and students' perception of classroom justice. Likewise, regarding the second research question, both teachers' immediacy and teachers' caring have been found to be the predictors of students' perception of classroom justice by employing SEM analysis. It can be noted that this finding was in line with the outcomes of Liu (2021) study underscoring the considerable role of teacher verbal and non-verbal immediacy on students' motivation and it was also consistent with Zheng (2021) emphasizing the impact of teacher clarity, immediacy, and credibility on students' motivation and classroom engagement even though students' perception of justice was not a variable in the above studies and instead, students' motivation and engagement were analyzed. Additionally, the findings of the current research are somewhat consistent with the following Derakhshan's (2021) in which it has been found that Turkman students' academic engagement is highly impacted by teachers' verbal and non-verbal immediacy. Alternatively, Gholamrezaee and Ghanizadeh (2018) found that students' constructs such as self-esteem and self-actualization are influenced by teachers' immediacy. Knesting's (2008) findings provide support for the present study in that it highlights how students' confidence, talent, and values are affected positively when they are treated justly by their teachers. Besides, in line with what Estaji and Zhaleh (2021) reported, the results of the present study corroborate that classroom justice plays a crucial role in the teachers' instructional practice.

## Conclusion

As it has been shown in the current study, teacher caring, teacher immediacy, and students' perception of classroom justice are positively correlated. Another result that was revealed in this study was that teacher caring and teacher immediacy predict students' perception of classroom justice. Two groups incorporating teacher educators and teachers themselves can be impacted by the results of this study. Without a shadow of a doubt, teachers should be equipped with theoretical and pedagogical knowledge as it empathetically affects teachers' physical and mental well-being (Dewaele and Dewaele, 2020). Therefore, in this regard, the results of this study give credence to teacher educators who are supposed to provide teachers with such knowledge discussed above. Moreover, teaching is not a one-dimensional job that is not demanding; it is a multidimensional career that takes perseverance and mental energy to create a learning context in which students can reach their pinnacle and high academic achievements can be achieved. Thus, teachers themselves are the second group that can benefit from this study since they deserve to feel good both about themselves and about their jobs. Working with students and having struggled to make them feel satisfied with what they have been taught and a learning context causes the teachers to feel tension and stressed. To relieve the tension, teachers should feel valued in order to boost their spirits, and it is not attainable unless teachers will come to the belief that their class and the way they teach and treat the students is perceived as fair in the eye of the students. The following examples are the actions that can be done by a teacher in the classroom so as to cause students to have a positive perception of classroom justice; however, it should be kept in mind that care should be taken to apply such activities in class because students' well-being in the class to a great extent rests on the following activities that can be utilized by the teachers:

- when a teacher makes a timely communication of his expectations with students at the beginning of the semester;
- when he provides the students with sufficient and honest information regarding the criteria that are employed in grading them;
- when the students are graded based on their achievements;
- when equal attention and help are provided to both high and low achievers in the class;
- when a teacher has a caring and supportive relationship with students;
- when a teacher is sensitive to his students' feelings, opinions, ad rights;
- when students are adequately informed of the class attendance policy if a topic has been taught incorrectly;
- when the teacher attempts to provide correct information subsequently;
- when equal opportunities and time are set for the students to participate in classroom discussions;
- when there is no favorite student in class to be treated differently from others in class;
- when students are allowed to express their concerns about the learning process.

The first limitation of this study that can be taken into consideration is that this study was cross-sectional and correlational; therefore, a longitudinal study can be implemented to provide teachers with more examples in detail since these characteristics for teachers help them create a living-learning environment for students in which students are encouraged to express themselves better and have a better understanding of justice. Moreover, the collected data in this study drawn from four comprehensive universities located in different cities in Henan Province. Great proportion of the sample students were from local areas and belonged to Han ethic group. The results of this study were applicable to central part of China, but cannot be blindly generalized to coastal areas or autonomous regions like Tibet or Ningxia. Future studies may be conducted to include more regions.

The second limitation is the amount of care and immediacy may differ considering the time. When it is at the beginning of the semester, students are more curious both about teachers' characteristics and about the subject-matter. As time goes by, they may get conditioned and habituated to the situation in which they are engaged to do the activities, their perception of classroom justice may change, and they may feel reluctant to consider teachers' caring and immediacy as positive since they need to be given a variety of activities so as to feel passionate about the learning process as time passes throughout the semester. Thus, there is a difference between how students feel about the class and their teacher at the beginning of the semester and the one which will be felt throughout the semester. In this regard, a longitudinal study appears of great importance. Student caring and teachers' willingness to teach better would be the title of another future study in which the focus will be shifted to students rather than teachers. The teacher-student relationship is reciprocal where both teachers and students should have enough enthusiasm to enhance this relationship although it is said that students are more impacted by their teachers and the way they are treated by their teachers in the classroom is of paramount significance especially when it comes to students' progress. Because teachers are in need of being cared for and being valued, students who are caring are perceived as encouraging for their teachers since they cause them to teach in an effective way. Another limitation of this study is the age through which justice is perceived by students is of great importance. It has been said that the older the students are, the wider their horizon would be which leads to students perceiving more and more about justice around them in the teaching context. Therefore, the definition of justice is different, from the prospect of various aging groups of students. Teenagers, for instance, may consider not running eyes over all the students equally as unfair, while adult students expect to be given enough care, otherwise, it makes them feel humiliated. Another concern can be the amount to which students are supposed to be given both verbal and non-verbal immediacy and caring. It is not proven that the more caring a teacher is, the more satisfied the students are regarding the learning process. So, more studies can be conducted in the future to find out the interplay of these variables with other teacher-student interpersonal factors (see Xie and Derakhshan, 2021).

## Data Availability Statement

The raw data supporting the conclusions of this article will be made available by the authors, without undue reservation.

## Ethics Statement

The studies involving human participants were reviewed and approved by Henan University Ethics Division. The patients/participants provided their written informed consent to participate in this study.

## Author Contributions

PY conceptualized, designed research methodology, collected data, analyzed data, and as well as independently drafted the manuscript.

## Funding

This study was supported by the Postgraduate Education Innovation Project of Henan University: Xenophobia—A discourse historical approach (Grant No. SYL20060127) and the Young Scholar Program of Henan Province: Foreign Language Education based on Virtual Reality Technology (Grant No. 2019GGJS036).

## Conflict of Interest

The author declares that the research was conducted in the absence of any commercial or financial relationships that could be construed as a potential conflict of interest.

## Publisher's Note

All claims expressed in this article are solely those of the authors and do not necessarily represent those of their affiliated organizations, or those of the publisher, the editors and the reviewers. Any product that may be evaluated in this article, or claim that may be made by its manufacturer, is not guaranteed or endorsed by the publisher.
